# Informed decision making for the elderly patient with newly diagnosed coeliac disease 

**Published:** 2020

**Authors:** Mohamed G Shiha, Lauren J Marks, David S. Sanders

**Affiliations:** *Academic Unit of Gastroenterology, Royal Hallamshire Hospital, Sheffield, United Kingdom*

**To The Editor**

We thank Dr. W.Shanahan and Dr. J.Shanahan for their interest in our study (1). In their letter, they have raised a few important points that we wish to clarify.

Firstly, they implied that we had a lack of appreciation for the recognition and prevention of bone-related morbidity in elderly Coeliac Disease (CD) patients. We respectfully disagree with their statement; throughout our paper, we repeatedly highlighted the importance of recognising such complications in this group of patients. Moreover, we reported that elderly CD patients had a significantly higher risk of osteopenia/osteoporosis compared to younger patients. In our practice, we routinely measure serum vitamin D, alkaline phosphatase and calcium levels for all CD patients and refer those older than 55 years or with known osteoporotic risk factors for bone density scans following the British Society of Gastroenterology guidelines. (2) A single centre audit, while valuable in improving local practices, should not be used to generalise poor adherence to national guidelines. 

Secondly, we agree that CD is associated with an increased risk of osteoporosis and fractures. However, GFD adherence rarely reverses bone loss in adults. This is particularly evident in elderly patients with long-term exposure to gluten and established bone disease at the time of diagnosis (3, 4). In our study, 57% of the osteopenic/osteoporotic elderly patients with CD were completely asymptomatic. Therefore, without strong evidence from randomised controlled trials or large prospective studies showing a clear benefit from adhering to a strict GFD over nutritional supplementation, only in this age group, we have to raise the question whether it is worthwhile breaking lifetime dietary habits in asymptomatic elderly patients.

Finally, should we be diagnosing Coeliac Disease in the elderly? The answer is probably yes. However, the majority of these elderley patients show little to no gastrointestinal symptoms and are often referred due to cancer or metabolic pathways with unexplained anaemia and bone loss. Hence, the diagnosis of CD may come as a shock to them. We feel that empowering elderly patients to adopt a GFD on an individual level based on the presence or absence of symptoms might be appropriate. Nonetheless, future research is needed to evaluate the alternatives to GFD and the other long-term risks of CD, such as malignancy and anaemia, before accepting such an approach. 
